# Development and validation of a quality of life measurement scale specific to hereditary hemorrhagic telangiectasia: the QoL-HHT

**DOI:** 10.1186/s13023-022-02426-2

**Published:** 2022-07-19

**Authors:** Thi Thao Truc Le, Guillaume Martinent, Sophie Dupuis-Girod, Antoine Parrot, Anne Contis, Sophie Riviere, Thierry Chinet, Vincent Grobost, Olivier Espitia, Brigitte Dussardier-Gilbert, Laurent Alric, Guillaume Armengol, Hélène Maillard, Vanessa Leguy-Seguin, Sylvie Leroy, Murielle Rondeau-Lutz, Christian Lavigne, Shirine Mohamed, Laurent Chaussavoine, Pascal Magro, Julie Seguier, Mallorie Kerjouan, Sylvie Fourdrinoy

**Affiliations:** 1 Laboratoire de Psychologie sur les Dynamiques Relationnelles et Processus Identitaires (EA 7458), Université de Bourgogne-Franche-Comté, 3 Allée des Stades Universitaires, 21000 Dijon, France; 2 Laboratoire sur les Vulnérabilités et l’Innovation dans le Sport (EA 7428), Université de Lyon, Université Claude Bernard Lyon 1, 27-29 bd du 11 Novembre 1918, 69622 Villeurbanne, France; 3 Service de génétique clinique, Centre de Référence pour la Maladie de Rendu-Osler, Hospices Civils de Lyon, HFME Bâtiment A1, 59 bd Pinel, 69677 Bron Cedex, France; 4 Service de pneumologie, Hôpital Tenon, Assistance Publique-Hôpitaux de Paris, 4 rue de Chine, 75790 Paris Cedex 20, France; 5 Service de médecine interne, Hôpital Saint André, 1 rue Jean Burguet, 33000 Bordeaux, France; 6 Service de médecine interne, CHU de Montpellier Hôpital St Eloi, Avenue A. Fliche, 34295 Montpellier Cedex 5, France; 7 Consultation Maladie de Rendu-Osler, CHU Ambroise Paré, 9 av Charles de Gaulle, 92104 Boulogne Billancourt, France; 8 Service de médecine interne, CHU Estaing, 1 rue Lucie et Raymond Aubrac, 63100 Clermont-Ferrand, France; 9 Service de médecine interne – médecine vasculaire, CHU de Nantes, 1 place Alexis Ricordeau, 44093 Nantes, France; 10 Service de génétique médicale, CHU de Poitiers, BP. 577, 86021 Poitiers Cedex, France; 11 Service de médecine interne, CHU Rangueil, 1 av du Pr Jean Poulhès, 31059 Toulouse Cedex 9, France; 12 Service de médecine interne, CHU de Rouen Ch. Nicolle, , 1 rue de Germont, 76031 Rouen Cedex, France; 13 Service de médecine interne, Hôpital Huriez, 1 rue Michel Polonovski, 59037 LILLE Cedex, France; 14 Service de médecine interne, Hôpital Le Bocage, 2 Bd Maréchal de Lattre de Tassigny, BP 77908, 21079 Dijon Cedex, France; 15 Service de pneumologie, CHU de Nice, 30 av de la Voie Romaine, 06002 Nice Cedex, France; 16 Service de médecine interne, CHU de Strasbourg Nouvel Hôpital Civil, 1 place de l’Hôpital, 67000 Strasbourg, France; 17 Service de médecine interne, CHU d’Angers, 4 rue Larrey, 49933 Angers Cedex 09, France; 18 Service de médecine interne, CHU de Nancy, Hôpital Brabois, Rue du Morvan, 54511 Vandoeuvre Les Nancy, France; 19 Service de médecine vasculaire, CHU de Caen, Avenue de la Côte de Nacre, 14000 Caen, France; 20 Service de pneumologie, CHRU de Tours Hôpital Bretonneau, 2 bd Tonnellé, 37044 Tours Cedex 9, France; 21 Service de médecine interne, Hôpital de La Timone, 264 rue Saint Pierre, 13385 Marseille Cedex 05, France; 22 Service de pneumologie, CHU de Rennes Hôpital Pontchaillou, 2 rue Henri Le Guilloux, 35033 Rennes Cedex 09, France

**Keywords:** Disease-specific scale, Hereditary hemorrhagic telangiectasia, HHT, Instrument development, Quality of life, Rendu–Osler disease

## Abstract

**Background:**

Hereditary hemorrhagic telangiectasia (HHT) disease is a rare genetic disorder with symptoms and complications that can significantly affect patients’ daily lives. To date, no scale has been validated to assess the specific symptoms of this disease on the quality of life (QOL) of HHT patients. This makes it difficult for clinicians to accurately measure the quality of life of patients with HHT. The present study aims to develop and validate a QOL measurement tool specific to HHT disease: the QOL questionnaire in HHT (QoL-HHT).

**Methods:**

A quantitative, non-interventional, multi-center study involving HHT patients in twenty French HHT expert centers was conducted. A calibration sample of 415 HHT patients and a validation sample of 228 HHT patients voluntarily participated in the study. Data were analyzed using exploratory factor analysis (EFA), confirmatory factor analysis (CFA), Exploratory Structural Equation Modeling (ESEM) analyses, reliability analyses, and correlational analyses.

**Results:**

The EFA, CFA and ESEM results allowed us to provide evidence of the factorial structure of a questionnaire composed of 24 items measuring 6 domains of QOL: Physical limitations, social relationships, concern about bleeding, relationship with the medical profession, experience of symptoms, and concern about the evolution of the disease. Cronbach’s alpha coefficients (> 0.70) demonstrated reliable internal consistency of all the QoL-HHT scores (dimensions). The results of the test–retest provided further evidence of the reliability of the QOL-HHT scores over time. Correlational analyses provided evidence for the convergent validity of the QoL-HHT scores.

**Conclusions:**

We developed a simple and quick self-assessment tool to measure quality of life specific to HHT disease. This study demonstrated reliability and validity of our QoL-HHT scores. It is a very promising tool to evaluate the impact of HHT disease on all aspects of the quality of life of HHT patients in order to offer them individualized medico-psycho-social support.

*Trial registration*: ClinicalTrials, NCT03695874. Registered 04 October 2018, https://www.clinicaltrials.gov/ct2/show/NCT03695874

**Supplementary Information:**

The online version contains supplementary material available at 10.1186/s13023-022-02426-2.

## Background

Hereditary hemorrhagic telangiectasia (HHT) is a rare but ubiquitous autosomal dominant genetic vascular disease with a prevalence of approximately 1/6000 births [1]. Also called Rendu–Osler disease in France, HHT is a pathology of angiogenesis deregulation leading to arteriovenous dilatations, hemorrhagic mucocutaneous telangiectasias and visceral shunts [2–4]. Symptoms vary between individuals but most of the time patients present with nasal bleeding (96% of patients), hemorrhages, mucocutaneous telangiectasias and visceral shunts by arteriovenous malformations [3–6]. Diagnosis can be done on complications related to malformations of the vessels of the lung, liver, brain and spinal cord [3–6]. These complications and symptoms significantly affect patients' daily life, social relationships, and professional life [7, 8].

QOL refers to an individual's sense of overall well-being, encompassing physical, psychological, emotional, and social dimensions, as a result of satisfaction or dissatisfaction in the areas of life that are important to them [9, 10]. QOL is not a representation of health on its own; while health can influence QOL and the impact of a health condition often results in a lower QOL, the concept of QOL represents the overall picture of well-being [10–12]. Clearly define the concept of QOL is a key point because the term is often inaccurately used to refer to a variety of related concepts that are by definition distinct from the construct of QOL [11]. Indeed, many health professionals use this term to refer to patient well-being in terms of health-related QOL, potentially far removed from the patient's overall perception [11].

Cohen and Biesecker [11] pointed out the importance of studying disease-related factors and especially psychosocial factors to understand what affects QOL of people living with rare genetic diseases. Many studies have shown that disease-related factors have a negative impact on the QOL of individuals with genetic diseases [8, 13]. This is especially the case for HHT patients. For example, a previous qualitative study of HHT patients showed that an important part of QOL was mainly determined by their health status and also revealed that the symptoms with the greatest impact on patients' daily life were epistaxis and fatigue [14]. Epistaxis (or nosebleeds) are the most frequent manifestation of HHT and are characterized by irregular, frequent, spontaneous, and mostly unpredictable occurrences [7]. Being the cause of iron deficiency and anemia, which can lead to significant fatigue in daily activities, nosebleeds create a concern about other people’s view [7, 14]. The diagnosis of HHT does not only refer to the physical consequences, but also questions the psychosocial dynamics inherent to this rare disease. Martinent et al. [14] showed that HHT affects the social and professional activities of patients and that communication about the disease and sharing of experiences within the family are sources of well-being. The medical care provided by health professionals also had an important impact on QOL [14]. A negative perception of one's own health and emotional consequences were also pointed out [8, 13].

A challenge in the field of QOL research refers to its measurement. The strategy to measurement and selection of a particular instrument is largely a function of the definition of QOL [11, 15]. There are two main approaches to measuring QOL: Using generic scales or using disease-specific scales. Many tools exist to measure it and most often focus on the physical, emotional, and social domains [11]. In a review of the literature on the measurement of QOL in rare diseases, the majority of studies use generic measurement scales [11]. In the case of HHT, Cohen and Biesecker report that 2 studies used the short form health survey (SF36) [16]. The first study showed that the QOL of patients with HHT was lower than that of controls and that its level was correlated with the intensity of physical symptoms [8]. The second study published similar results, highlighting the importance of perceived symptom consequences [13]. In a more recent study focused on HHT, Zarrabeitia et al. [17] also used a generalist scale, the Euro quality of life 5 dimensions 3 level version (Euroqol-5D-3L) [18] supplemented with a subjective self-report of nosebleeds (mild, moderate, or severe). Their results showed higher scores on all five dimensions of the EuroQol 5D-3L in people with HHT disease than in the general population, particularly with regard to pain, anxiety, discomfort and depression [17]. Generic scales therefore have the advantage that they can be used regardless of the pathology presented by the patients and allow comparisons between pathologies. However, they have the disadvantage of not taking into account the particularities of the pathologies and their symptoms, of lacking sensitivity when one wishes to evaluate the evolution of the QOL over a given period of time and of not considering the importance attributed by patients to a specific domain of the QOL [19]. Generic scales therefore do not provide a detailed and precise view of the problems encountered by patients within particular disease and their impact on their QOL.

The patient cohort followed by the French HHT network (Reference Center and Competence Centers) reaches about 4000 cases. During outpatient follow-up, patients systematically talk with their doctor about the impact of the disease on their daily life. [14]. However, the tools currently available to adapt the management of patients do not satisfy the evaluation of the impact of the specific symptoms of HHT on the QOL. While specific scales for measuring QOL exist for rare diseases such as cystic fibrosis or sickle cell disease [20, 21], no scale to date has been validated for HHT disease. Based on a previous qualitative study [14] that has examined patients' subjective experience, their problems and their representations of the QOL our objective is therefore to develop a QOL measurement tool specifically adapted to HHT disease.

## Methods

### Participants

This national, quantitative, non-interventional, multi-center study involved twenty French HHT expert centers. The calibration sample included a total of 415 HHT patients and the validation sample included a total of 228 HHT patients. Participants were selected according to the following inclusion criteria: (a) persons with clinically and/or genetically confirmed HHT disease [22], (b) persons with 18 years of age or older and (c) persons who are fluent in French.

### Development of the preliminary version of the QoL-HHT

A preliminary phase was carried out using a qualitative method; its aim was to understand the complexity of the experience of the disease and the subjectivity of the people interviewed [14]. The object of this qualitative study was to bring out the specificity of the daily impact of a disease for which there is a double specificity of being genetic and rare compared to more "common" diseases. Following this qualitative study, an expert panel consisting of five researchers and/or doctors was composed. The members of the panel are experts in HHT or expert in questionnaire construction, as they work with HHT patients or have been involved in and published peer-reviewed articles in questionnaire validation, respectively. Based on the aforementioned qualitative study conducted with HHT patients [14], an initial pool of 78 items was created by the panel. These 78 items cover the 6 categories emerging within this study: The impact of physical symptoms on daily life, quality of family and social life, emotional and psychological outcomes related to the disease, knowledge of having a severe disease and coping strategies to manage such disease, recognition of the disease by professional colleagues and superiors, and knowledge and understanding from health professionals in medical care [14]. Thus, these items were specific to HHT and spoke to the patients' subjective experience. They dealt in particular with the consequences of fatigue, which are not covered by the generic scales, knowledge and recognition of the disease by those around them in the broad sense (family, friends, professionals, doctors, social workers), and the question of transmission, a subject specific to genetic diseases. A pre-test phase was carried out with 10 HHT patients in order to ensure of the understanding and the clarity of each of the 78 preliminary items (leading to the rewording of some items). A 5-point Likert scale ranging from 1 (strongly disagree) to 5 (strongly agree) was used.

### Questionnaires used for convergent validity of the QoL-HHT scores

A battery of questionnaires was used to test the convergent validity of the QoL-HHT scores): Short-Form 36 (SF36) [23], Social Support Questionnaire 6 (SSQ6) [24], Cognitive Emotional Regulation Questionnaire (CERQ) [25] and Hospital Anxiety and Depression (HAD) [26].

The SF36 was developed as part of the Medical Outcome Study [27]. The French version [23] of this scale contains thirty-six items exploring eight different domains: (1) limitations in physical activities related to health problems, (2) limitations in social activities related to physical or emotional problems, (3) limitations in usual role, (4) bodily pain, (5) general mental health, (6) limitations in usual activities related to emotional problems, (7) vitality (energy and fatigue), and (8) perceived general health. Responses are either binary (yes/no) or scaled in three to six points. This scale gives two scores, one for physical health and the other for psychological health. The results from the "vitality" and "general health" domains are integrated simultaneously into the two scores. Each dimension is scored from 0 to 100: the higher the score, the better the QOL. This scale can be self-administered or collected by an interviewer, which takes 5 to 10 min.

The French version [24] of the short form of the social support questionnaire [28] was used to assess perceived social support. Two dimensions of social support were assessed: Availability (number of people available to support the subject) and satisfaction (is this support satisfactory?). Six items evaluated each dimension. For availability, participants reported the number of perceived social support sources from none to nine individuals. For satisfaction, respondents reported how satisfied they are with the received support on a 6-point Likert scale ranging from 1 (very dissatisfied) to 6 (very satisfied). As such, the scores of the satisfaction scale vary from 6 to 36.

The French version [25] of the CERQ [29] measures the prevalence of various cognitive emotional regulation strategies to cope with unpleasant situations. In particular, the subject is asked to reflect on the way he/she thinks in general when confronted with negative or unpleasant events. This scale thus made it possible to characterize the regulation style of an individual when faced with negative events [30]. This scale includes nine 4-item sub-dimensions: Self-blame, blame others, acceptance, action focus, positive focus, rumination, positive reappraisal, perspective-taking and finally, dramatization. Participants responded on a 5-point Likert scale ranging from 1 (almost never) to 5 (most of the time).

The French version [26] of the HAD [31] was used to assess anxiety (HAD-A subscore; 7 items) and depression (HAD-D subscore; 7 items). It consists of 14 items evaluated using a 4-point Likert scale ranging from 0 (never) to 3 (most of the time). Thus, the two scores (anxiety and depression) ranged from 7 to 21. The threshold of 8 is used for each sub-score to detect anxiety or depression symptoms. In particular, the different levels of depression or anxiety correspond to mild (score 8–10), moderate (11–14) and severe (15 and above) [32]. As such, a score of 7 or less (on each of the dimension) refers to the absence of symptomatology.

### Procedure

The patients were recruited: (a) during their consultation in one of the twenty French HHT expert centers, (b) during medical days organized by the AMRO-HHT-France (Rendu–Osler Disease patients Association—HHT) or (c) following a proposal by mail (electronic or postal) of the medical center ensuring their follow-up. The patients were informed of the study by the investigating physicians and indicated that they had no objection to their participation. The research recorded on ClinicalTrials.gov (NCT03695874) was conducted in accordance with international ethical guidelines that are consistent with American Psychological Association norms and was approved by the local research ethics committee (Comité de Protection des Personnes Sud-Est III: n°2018-A02128-47).

Participants of the calibration sample completed anonymously the preliminary version of the QoL-HHT questionnaire (78-item version). Participants of the validation sample completed anonymously the 24-item version (i.e., a shortened version of the QoL-HHT questionnaire) resulting from the Exploratory Factorial Analyses (EFA) as well as other questionnaires used for the convergent validity of the QOL-HHT scores: SF36 [23], SSQ6 [24], CERQ [25], and HAD [26]. Finally, over the 228 patients of the validation sample, 136 patients also completed the QoL-HHT questionnaire a second time one month after the first completion. These data were used for the test–retest.

### Data analysis

The population of 415 patients (calibration sample) was used to perform Exploratory Factorial Analyses (EFA) with the preliminary version of the 78-item QoL-HHT questionnaire. In particular, a series of EFAs with varimax rotation using maximum likelihood extraction was used to extract factors of the calibration data to determine the appropriate number of factors in the scale development procedure [33]. The number of factors was determined by examining a visual inspection of the scree plots with the criterion of eigenvalues greater than 1 [34]. Then, an iterative approach was conducted in which problematic items were successively deleted based on several statistical criteria: (1) factor loadings on the main factor must be greater than 0.40, and (2) items with several factor loadings greater than 0.40 (on different factors) were deleted (i.e., one item must belong to only one factor) [35]. Because saturations can change with the addition or removal of items, the remaining items were subjected to iterative follow-up EFAs until all such items were identified and removed. These analyses were rerun each time an item was removed until all remaining items fully met the statistical and clinical criteria set. The clinical criteria refers to the content (meaning) of the items. In particular, we ensured that the several items loading on a particular factor shared themes to ensure that the obtained factors can be understandable from a theoretical point of view.

Because the model generation strategy used in the re-estimation of the QoL-HHT through item deletion could be sensitive to chance capitalization [36], the final shortened version of the questionnaire resulting from the EFAs was further evaluated by fitting it to an independent validation sample composed of 228 HHT patients using the Confirmatory Factorial Analysis (CFA), the Exploratory Structural Equation Modeling (ESEM), and bifactor models. All models were estimated using Mplus 7.3 [37] and a robust maximum likelihood estimator. The CFA model was specified according to theory expectations: Each item loaded on the target factor and all cross-loadings (not belonging to the latent dimension under consideration) were computed to be zero. For the bifactor model, each item was specified as loading on a general factor QOL factor as well as on their specific factors, corresponding to the six distinct dimensions of patient QOL identified within the EFAs. Finally, the ESEM model allowed for the estimation of cross-saturation coefficients not belonging to the latent dimension under consideration. These non-target latent factor coefficients are assumed low (and as close to zero as possible) but are freely estimated at non-zero values [38]. For the CFA, bifactor and ESEM model, model fit was assessed using several fit indices: the chi-square (χ^2^), comparative fit index (CFI), Tucker–Lewis index (TLI), root mean square error of approximation (RMSEA) with its confidence interval (90% CI), standardized root mean square residual (SRMR), Akaike Information Criteria (AIC), Bayesian Information Criteria (BIC), and sample size-adjusted BIC (ABIC). CFI and TLI values greater than 0.90 refer to an acceptable fit and values greater than 0.95 refer to an excellent fit to the data. RMSEA and SRMR values below 0.08 refer to an acceptable fit model and values below 0.05 refer to a model with an excellent fit to the data collected. AIC, BIC, and ABIC were also used to compare the models to each other. Lower values of AIC, BIC, and ABIC indicate a better fit to the collected data.

Third, the reliability of QoL-HHT scores was assessed using Cronbach's alpha coefficients and composite reliability values (ρ). Composite reliability values (i.e., ρ = [(sum of standardized loadings)^2^]/[(sum of standardized loadings)^2^ + (sum of error variances)]) measure the overall reliability of a collection of heterogeneous but similar items [39]. A value of 0.70 or greater indicates an acceptable reliability [39]. Fourth, test-retests of the QoL-HHT subscales were computed over a one-month period.

Fifth, correlations between the QoL-HHT subscales and the four external scales (SF36, SSQ6, CERQ and HAD) were used to examine the relationships between the QoL-HHT scores and the other external variables. Correlations were interpreted using Cohen’s criteria [40] (small ≤ 0.30; medium = 0.30 to 0.50; large ≥ 0.50).

## Results

415 HHT patients were included in the calibration sample, 266 women and 149 men with an average age of 52 years (± 16.2). The validation sample included a total of 228 HHT patients, including 147 women and 78 men with an average age of 53 years (± 16.5).

### Initial EFA and follow-up EFAs on the calibration sample

The scree test provided strong evidence of a six-factor solution (and all the six first eigenvalues were higher than 2). However, several items did not achieve a loading of 0.40 on any factor, whereas other items loaded on several factors simultaneously (see additional file 1 for more details). We therefore re-estimated the calibration model by systematic and sequential item deletion, resulting in a final solution of 29-items six factors. This process resulted in the removal of 49 items. The scree test also provided strong evidence of six-factor solution with the 29 remaining items, with only six eigenvalues higher than 1 (6.87, 3.84, 1.97, 1.78, 1.59, 1.47) (data available from the first author). Factor 1 included 6 items assessing physical limitations, particularly physical fatigue. Factor 2 included 4 items assessing social relationships and referred to relationships with family and friends. Factor 3 included 4 items assessing concern about bleeding. Factor 4 included 6 items assessing the relationship with the medical profession, including advice given by caregivers. Factor 5 included 5 items assessing concern about symptoms, particularly in patients' daily lives. Finally, factor 6 included 4 items assessing concern about the evolution of the disease, particularly with regard to its evolution over time and the hereditary nature of the disease. Higher scores on factors 2, 4, and 5 reflected a more positive assessment or experience of the circumstances, while higher scores on factors 1, 3, and 6 reflected a more negative assessment or experience of the circumstances. Factor loadings of the final 29-items 6-factors EFA model of the calibration sample are presented in Table 1.

**Table 1:** Factor Loadings, Eigenvalues, Percentage of variance, and Internal Consistency of EFAs for the calibration sample

Items	Factor 1	Factor 2	Factor 3	Factor 4	Factor 5	Factor 6
*Factor 1: PL*						
Item 1	**0.62**	− 0.04	0.19	− 0.03	− 0.21	.012
Item 2	**0.85**	0.01	0.16	− 0.01	− 0.09	0.12
Item 3	**0.76**	− 0.05	0.23	0.06	− 0.17	0.00
Item 4	**0.78**	0.02	0.28	− 0.03	− 0.14	0.07
Item 5	**0.84**	0.05	0.17	− 0.07	− 0.06	0.10
Item 6	**0.77**	− 0.03	0.09	0.01	0.04	0.06
*Factor 2: SR*						
Item 7	0.00	**0.69**	0.11	0.22	0.02	0.04
Item 8	0.08	**0.71**	− 0.03	0.12	0.15	− 0.07
Item 9	− 0.05	**0.80**	0.05	0.15	0.10	− 0.01
Item 10	− 0.01	**0.77**	− 0.16	0.04	0.09	0.02
*Factor 3: CAB*						
Item 11	0.20	− 0.03	**0.84**	− 0.02	− 0.01	0.11
Item 12	0.39	− 0.05	**0.68**	0.02	− 0.11	0.09
Item 13	0.25	− 0.03	**0.83**	− 0.03	− 0.06	0.14
Item 14	0.15	0.06	**0.82**	0.05	− 0.10	0.11
*Factor 4: RMP*						
Item 15	− 0.15	0.12	− 0.13	**0.73**	0.17	0.02
Item 16	0.01	0.12	− 0.11	**0.75**	0.02	0.01
Item 17	0.12	0.15	0.08	**0.53**	0.23	− 0.14
Item 18	− 0.23	0.20	0.05	**0.61**	0.15	− 0.06
Item 19	0.20	0.02	0.13	**0.65**	− 0.15	− 0.03
Item 20	− 0.01	0.23	0.15	**0.62**	0.12	0.13
*Factor 5: ES*						
Item 21	0.05	0.02	− 0.27	0.09	**0.61**	0.05
Item 22	− 0.18	0.15	− 0.12	0.23	**0.64**	− 0.07
Item 23	− 0.31	0.05	− 0.08	0.10	**0.64**	− 0.11
Item 24	− 0.13	0.09	− 0.06	0.08	**0.74**	− 0.06
Item 25	− 0.17	0.28	0.01	− 0.07	**0.65**	− 0.18
*Factor 6: CED*						
Item 26	0.28	0.05	0.25	− 0.14	− 0.11	**0.60**
Item 27	0.01	− 0.09	0.05	0.19	0.08	**0.71**
Item 28	0.11	0.02	0.10	0.03	− 0.14	**0.76**
Item 29	0.21	0.01	0.33	− 0.13	− 0.14	**0.70**
Eigenvalues	6.87	3.84	1.97	1.78	1.59	1.47
Percentage of variance	23.70	13.25	6.79	6.14	5.47	5.07
Internal consistency	0.90	0.77	0.87	0.77	0.74	0.73

Boldface indicates representative factor loadings

*PL* Physical limitations; *SR* Social relationships; *CAB* Concern about bleeding; *RMP* Relationship with the medical profession; *ES* Experience of symptoms; *CED* Concern about the evolution of the disease

### CFA, bifactor and ESEM on the validation sample

The goodness-of-fit indices for the CFA, ESEM, and bifactor models were not acceptable for the 29-item version of the questionnaire (see additional file 2 for more details) because some goodness-of-fit scores did not reach the criterion-specific acceptability threshold and some standardized factor loadings were lower than 0.40. In particular, the poor goodness of fit indices of the bifactor model (CFI = 0.83, TLI = 0.80, SRMR = 0.247) clearly provided evidence against the existence of a general dimension of QOL.

Therefore, we used an iterative process on the CFA model, where items were removed one by one on the basis of the statistical considerations mentioned above (i.e., standardized factor loading higher than 0.40) in order to obtain an acceptable factor structure of the QoL-HHT questionnaire. Following this procedure, 5 items (items 1, 6, 17, 19, 21) with low factor loadings were deleted and resulted in a final version of the QoL-HHT questionnaire with a total of 24 items including 4 items for each of the six factors. The goodness-of-fit indices of the CFA 24-item six-factor correlated model and 24-item six-factor ESEM model reached cut-off criterion values for an acceptable fit to the data (Table 2). All standardized factor loadings of the CFA and ESEM models (for the target factor) were significant at *p* < 0.05 and were higher than 0.40 (Table 3; items have been translated in English for a better understanding of this table). In particular, the standardized factor loadings for all the items ranged from 0.48 to 0.94 for the CFA model and ranged from 0.43 to 0.94 for the ESEM model. In addition, no cross-loadings were identified within the ESEM model for the not targeted items. Concerning the bifactor model, despite acceptable fit indices to the data for the 24-item QoL-HHT questionnaire (except for the SRMR value which does not reach the acceptability threshold), the results of the standardized factor loadings confirmed the results of the 29-item questionnaire and provided further evidence against the existence of a general factor of QOL (see additional file 3 for more details).

**Table 2:** Fit Indices of the CFA, ESEM, and Bifactor models for the validation sample

Model	χ^2^	df	CFI	TLI	AIC	BIC	ABIC	RMSEA	90%CI RMSEA	SRMR
CFA (29 items)	680.115	362	0.864	0.847	18229.776	18579.569	18256.298	0.062	0.055–0.069	0.079
ESEM (29 items)	446.051	247	0.915	0.860	18154.362	18898.530	18210.788	0.059	0.051–0.068	0.035
Bifactor (29 items)	731.044	340	0.833	0.800	18295.995	18721.234	18328.238	0.071	0.064–0.078	0.247
CFA (24 items)	405.033	237	0.910	0.895	15248.885	15547.238	15271.507	0.056	0.046–0.065	0.061
ESEM (24 items)	264.602	147	0.937	0.882	15245.482	15852.476	15291.507	0.059	0.048–0.071	0.032
Bifactor (24 items)	399.111	220	0.904	0.880	15271.481	15628.133	15298.524	0.060	0.050–0.069	0.169

*CFA* Confirmatory factor analysis; *ESEM* Exploratory structural equation modeling; χ^2^ = Chi-square; *df.* degrees of freedom; *CFI* Comparative fit index; *TLI* Tucker-Lewis index; *AIC* Akaike information criterion; *BIC* Bayesian information criteria; *ABIC* sample size-adjusted BIC; *RMSEA* Root mean square error of approximation; *SRMR* Standardized root mean square residual

**Table 3:** Standardized Factor Loadings (λ) and Uniqueness (δ) of CFA and ESEM models for the validation sample for the questionnaire with 24 items (items have been translated in English for a better understanding of this table)

	Confirmatory factor analysis		Exploratory structural equation modelling						
Items ^a^	λ	δ	PL (λ)	SR (λ)	CAB (λ)	RMP (λ)	ES (λ)	ED (λ)	δ
*Factor 1: physical limitations (PL)*									
1. I feel like I'm running in slow motion^a^	**0.79**	0.38	**0.77**	− 0.02	0.16	− 0.17	− 0.07	0.12	0.33
6. Hereditary Hemorrhagic Telangiectasia disease limits my movements (walking, mobility…)	**0.78**	0.39	**0.70**	0.09	0.22	0.12	− 0.25	0.06	0.37
12. Because of the disease, I am limited in my intense physical activities (running, heavy lifting, sports, etc.)	**0.78**	0.39	**0.71**	0.09	0.26	0.05	− 0.22	− 0.04	0.37
17. I am often physically tired	**0.76**	0.43	**0.80**	− 0.06	0.09	− .12	0.02	0.22	0.29
*Factor 2: social relationships (SR)*									
2. Being able to talk about the disease with my family helps me cope better	**0.58**	0.67	− 0.09	**0.58**	0.06	0.11	− 0.08	0.07	0.64
7. I can cope better with the disease thanks to the support of my friends	**0.55**	0.70	0.05	**0.49**	0.07	0.27	− 0.02	− 0.02	0.68
13. The fact that my family understands what I need improves my daily life	**0.83**	0.31	0.04	**0.75**	0.07	0.25	0.10	0.09	0.34
18. The fact that my family understands what I am going through makes my life better	**0.81**	0.34	0.06	**0.83**	0.07	0.14	0.07	0.03	0.28
*Factor 3: concern about bleeding (CAB)*									
3. I am bothered by the sudden and unpredictable nature of the bleeding	**0.63**	0.60	0.20	0.03	**0.54**	0.01	− 0.13	0.22	0.60
8. The frequency, duration and/or intensity of bleeding is very disturbing to me	**0.60**	0.64	0.37	− 0.03	**0.50**	0.01	− 0.21	0.12	0.56
14. I am often apprehensive about bleeding in public	**0.92**	0.16	0.17	0.14	**0.85**	− 0.06	− 0.18	0.13	0.19
19. I am very embarrassed to bleed in public	**0.94**	0.12	0.10	0.06	**0.94**	− 0.04	− 0.12	0.19	0.05
*Factor 4: Relationship with the medical profession (RMP)*									
4. Thanks to the medical advice offered to me, my quality of life is preserved	**0.74**	0.46	− 0.14	0.30	− 0.05	**0.63**	0.17	0.07	0.47
9. Thanks to medical recommendations, I can limit the impact of symptoms on my daily life	**0.74**	0.45	− 0.01	0.17	− 0.06	**0.81**	0.08	− 0.01	0.31
20. The quality of the relationships I have with the caregivers allows me to be serene	**0.58**	0.67	0.02	0.17	− 0.04	**0.45**	0.28	− 0.02	v.68
24. Thanks to the information given by the caregivers I can learn about the disease and get involved in the care	**0.61**	0.63	− 0.05	0.18	0.03	**0.54**	0.13	0.06	0.66
*Factor 5: experience of symptoms (ES)*									
10. The symptoms of the disease do not bother me too much because I have learned to manage them	**0.60**	0.64	− 0.18	0.03	− 0.18	0.24	**0.45**	− 0.18	0.64
15. Hereditary Hemorrhagic Telangiectasia disease seems so familiar to me that I don't feel like I'm sick	**0.79**	0.37	− 0.22	0.04	− 0.18	0.14	**0.69**	− 0.20	0.38
21. I don't feel embarrassed by the disease because I feel like I have always lived with it	**0.82**	0.33	− 0.10	0.05	− 0.21	0.16	**0.75**	− 0.19	0.32
23. Even though I often have nosebleeds, I don't pay much attention to it because it has become a habit	**0.64**	0.59	− 0.10	0.00	− 0.13	0.07	**0.64**	− 0.07	0.56
*Factor 6: Evolution of the disease (ED)*									
5. I fear that the disease will worsen with age	**0.69**	0.52	0.18	− 0.04	0.24	0.00	− 0.20	**0.59**	0.52
11. I am concerned about the health of other family members who have Hereditary Hemorrhagic Telangiectasia disease	**0.48**	0.77	− 0.01	0.18	0.15	0.03	− 0.19	**0.43**	0.73
16. I worry about my health when I see others around me who have Hereditary Hemorrhagic Telangiectasia disease	**0.57**	0.68	0.06	0.18	0.18	0.05	− 0.13	**0.53**	0.63
22. I am concerned about the evolution of the disease in the future	**0.78**	0.39	0.22	− 0.04	0.24	0.02	− 0.19	**0.68**	0.40

Boldface indicates representative factor loadings (*p* < .05)

*CFA* Confirmatory factor analysis; *ESEM* Exploratory structural equation modeling

^a^ English version of the items is provided for informational purposes and has not been subject to validation

### Reliability and test–retest

Cronbach’s alpha coefficients of the final sample (validation sample) ranged from 0.73 to 0.87 and ρ values ranged from 0.73 to 0.86, indicating that the reliability of each of the six QoL-HHT scores was acceptable (Table 4). Test-retests over a one month period revealed significant correlations (all *p*_s_ < 0.001), of 0.87, 0.48, 0.73, 0.46, 0.64 and 0.49 for QoL-HHT dimensions of physical limitations, social relationships, concern about bleeding, relationship with the medical profession, experience of symptoms and concern about the evolution of the disease, respectively.

**Table 4:** Descriptive statistics, reliability scores and correlations for the validation sample

Subscales	PL	SR	CAB	RMP	ES	ED	*α*
**QoL-HHT scores**							
Physical limitations (PL)	–						0.86
Social relationships (SR)	0.05	–					0.77
Concern about bleeding (CAB)	0.45***	0.12	–				0.87
Relationship with medical profession (RMP)	− 0.12	0.43***	− 0.08	–			0.76
Experience of symptoms (ES)	− 0.37***	0.08	− 0.43***	0.33***	–		0.80
Concern about the evolution of the disease (ED)	0.31***	0.14*	0.45***	0.01	− 0.39***	–	0.73
**SF36 scores**							
*Physical health score*							
Physical functioning	− 0.62***	− 0.08	− 0.26***	0.02	0.23***	− 0.19**	0.92
Role-physical	− 0.71***	− 0.08	− 0.34***	0.10	0.31***	− 0.28***	0.82
Bodily pain	− 0.55***	0.02	− 0.31***	0.09	0.17*	− 0.15*	0.86
General health	− 0.61***	− 0.02	− 0.34***	0.14*	0.44***	− 0.41***	0.85
*Psychological health score*							
Social functioning	− 0.50***	− 0.05	− 0.28***	0.11	0.36***	− 0.21**	0.66
Role-emotional	− 0.52***	− 0.01	− 0.26***	0.11	0.28***	− 0.22**	0.84
Mental Health	− 0.41***	0.09	− 0.27***	0.23***	0.27***	− 0.20**	0.80
Vitality	− 0.66***	0.08	− 0.38***	0.27***	0.31***	− 0.18**	0.84
**SSQ6 scores**							
Social availability	− 0.23***	0.18**	− 0.14*	0.11	0.08	0.01	0.88
Social satisfaction	− 0.12	0.08	− 0.03	0.16*	0.12	− 0.04	0.96
**CERQ scores**							
Self-blame	0.04	− 0.09	0.02	− 0.14*	− 0.04	0.14*	0.70
Acceptance	0.15*	0.09	− 0.01	0.11	0.03	0.08	0.72
Rumination	0.26***	0.10	0.14*	− 0.08	− 0.18**	0.15*	0.74
Positive focus	− 0.04	0.25***	− 0.05	0.33***	0.19**	− 0.10	0.88
Focus on action	− 0.13	0.17*	0.03	0.25***	0.14*	− 0.03	0.83
Positive reappraisal	− 0.21**	0.20**	− 0.16*	0.20**	0.25***	− 0.15*	0.84
Putting it into perspective	− 0.10	0.20**	− 0.05	0.24***	0.19**	− 0.05	0.77
Dramatization	0.36***	0.05	0.17*	− 0.08	− 0.15*	0.26***	0.72
Blame others	0.09	0.03	0.01	− 0.09	0.03	0.13	0.69
**HAD scores**							
Anxiety	0.20**	− 0.06	0.14*	− 0.25***	− 0.16*	0.13	0.70
Depression	0.55***	− 0.09	0.27***	− 0.31***	− 0.28***	0.12	0.81
*Mean*	2.99	3.42	3.82	3.58	3.23	3.89	
*Standard Deviation*	1.20	0.88	1.12	0.74	1.02	0.84	
*ρ values*	0.86	0.79	0.86	0.76	0.81	0.73	

^*^ *p* < 0.05; ** *p* < 0.01; *** *p* < 0.001

### Correlational analysis

Results of the correlational analysis (Table 4) showed that: (a) QoL-HHT inter-correlations ranged from − 0.43 to 0.46, suggesting that the six QoL-HHT subscales are tapping unique yet correlated dimensions of QOL; (b) QoL-HHT physical limitations was significantly positively correlated with acceptance (*r* = 0.15), rumination (*r* = 0.26), dramatization (*r* = 0.36), anxiety (*r* = 0.20) and depression (*r* = 0.55), and significantly negatively related to physical functioning (*r* = − 0.62), role-physical (*r* = − 0.71), bodily pain (*r* = − 0.55), general health (*r* = − 0.61), social functioning (*r* = − 0.50), role-emotional (*r* = − 0.52), mental health (*r* = − 0.41), vitality (*r* = − 0.66), social availability (*r* = − 0.23) and positive reappraisal (*r* = − 0.21); (c) QoL-HHT social relationships was significantly positively related to social availability (*r* = 0.18), positive focus (*r* = 0.25), focus on action (*r* = 0.17), positive reappraisal (*r* = 0.21) and putting it into perspective (*r* = 0.20); (d) QoL-HHT concern about bleeding was significantly positively correlated with rumination (*r* = 0.14), dramatization (*r* = 0.17), anxiety (*r* = 0.14) and depression (*r* = 0.27), and significantly negatively related to physical functioning (*r* = − 0.26), role-physical (*r* = − 0.34), bodily pain (*r* = − 0.27), general health (*r* = − 0.38), social functioning (*r* = − 0.28), role-emotional (*r* = − 0.26), mental health (*r* = − 0.27), vitality (*r* = − 0.38), social availability (*r* = − 0.14), and positive reappraisal (*r* = − 0.16); (e) QoL-HHT relationship with medical profession was significantly positively related to general health (*r* = 0.14), mental health (*r* = 0.23), vitality (*r* = 0.27), social satisfaction (*r* = 0.16), positive focus (*r* = 0.33), focus on action (*r* = 0.25), positive reappraisal (*r* = 0.20) and putting it into perspective (*r* = 0.24), and significantly negatively related to self-blame (*r* = − 0.14), anxiety (*r* = − 0.25) and depression (*r* = − 0.31); (f) QoL-HHT experience of symptoms was significantly positively correlated with physical functioning (*r* = 0.23), role-physical (*r* = 0.31), bodily pain (*r* = 0.17), general health (*r* = 0.44), social functioning (*r* = 0.36), role-emotional (*r* = 0.28), mental health (*r* = 0.27), vitality (*r* = 0.31),, positive focus (*r* = 0.19), focus on action (*r* = 0.14), positive reappraisal (*r* = 0.25) and putting it into perspective (*r* = 0.19), and significantly negatively related to rumination (*r* = − 0.18), dramatization (*r* = − 0.15), anxiety (*r* = − 0.16) and depression (*r* = − 0.28); (g) QoL-HHT concern about the evolution of the disease was significantly positively correlated with self-blame (*r* = 0.14), rumination (*r* = 0.15) and dramatization (*r* = 0.26), and significantly negatively related to physical functioning (*r* = − 0.19), role-physical (*r* = − 0.28), bodily pain (*r* = − 0.15), general health (*r* = − 0.41), social functioning (*r* = − 0.21), role-emotional (*r* = − 0.22), mental health (*r* = − 0.20), vitality (*r* = − 0.18) and positive reappraisal (*r* = − 0.15).

### Comparison between sexes and correlations with age

The results of the one-way ANOVA analysis (Table 5) showed no gender difference in the 6 dimensions of QOL (*ρ* > 0.05). Only the dimension of relationship with medical profession was close to being significant by gender (*ρ* = 0.07). Women had a higher mean score (Mean(F) = 3.65) than men (Mean(M) = 3.46) on the dimension of relationship with medical profession. Regarding the correlations (Table 5) between age and the different dimensions, the results showed significantly and positively correlations between age and physical limitations (*r* = 0.33) and concern about bleeding (*r* = 0.18).

**Table 5:** Descriptive statistics, comparison (Welch’s One-Way Anova) between women and men and correlations with age for the validation sample

Comparison between sexes						Correlations with age
	Sexe	Mean	Standard deviation	F	ρ values	Age
PL	F	3.02	1.23	0.38	0.54	0.33***
	M	2.92	1.17			
SR	F	3.49	0.90	2.35	0.13	− 0.08
	M	3.30	0.84			
CAB	F	3.85	1.07	0.32	0.57	0.18**
	M	3.76	1.17			
RMP	F	3.65	0.73	3.30	0.07	0.08
	M	3.46	0.76			
ES	F	3.31	1.01	1.58	0.21	− 0.09
	M	3.13	1.02			
ED	F	3.89	0.87	0.10	0.75	0.08
	M	3.86	.78			

F = Women; M = Men

** *p* < 0.01; *** *p* < 0.001

## Discussion

The objective of the present research was to develop and validate a QOL measurement tool specifically adapted to HHT disease – the QoL-HHT (Quality of Life—Hereditary Hemorrhagic Telangiectasia): A simple and quick tool to fill in for self-assessment. The results of the factorial structure, (Exploratory Factorial Analysis EFAs, Confirmatory Factorial Analysis CFA, Exploratory Structural Equation Modeling ESEM) reliability, and convergent validity of the QoL-HHT scores provided strong evidence of construct validity, indicating that the QoL-HHT is a promising scale developed for and in collaboration with HHT patients to allow assessment of the impact of HHT on the QOL of patients. The salient factors emerging from the factorial analyses of QoL-HHT scores referred to physical limitations, social relationships, concern about bleeding, relationship with the medical profession, experience of symptoms and concern about the evolution of the disease. Indeed, QoL-HHT scores (24 items) fitted well with CFA and ESEM models. The pattern of cross-loadings and the standardized factor loadings of the six subscales of the QoL-HHT provided evidence for the structural validity of the QoL-HHT scores. Test-retests provided evidence for the reliability and stability of the QoL-HHT scores over a one-month period. The six subscales would allow example the relationships between specific symptoms related to QOL of HHT patients and other theoretically-relevant constructs or outcomes, as they provide an in-depth assessment of the multidimensional construct of QOL with six interrelated but distinct dimensions specific to the HHT disease. The bifactor model did not allow us to provide evidence for an overall QOL score as the factorial structure of the QoL-HHT scores did not fit to a bifactor structure.

The physical limitations dimension refers to the physical consequences of the disease. The consequences of physical symptoms can result in significant fatigue in daily activities, especially at work, limiting physical activities [7]. The literature highlighted that the physical factor is inherent to most of the genetic diseases and is a salient dimension of the QOL of individuals with genetic diseases [8, 11, 13]. The salient consequences of the physical domain allow understanding the experiences of people with HHT disease and provides further evidence of the importance of this theme within the HHT disease [7, 14].

The concern about nosebleeds dimension refers to the experience of the most frequent symptom of the disease. Recurrent epistaxis is the most common presentation of HHT and has a large impact on patient QOL [41]. This experience is often marked by an uncertainty about the onset of epistaxis, which can occur at any time, thus creating anxiety in the eyes of others. It can also lead to feelings of insecurity and loss of confidence in one’s body, which sometimes make patients feeling helpless and out of control [14, 42]. Thus, episodes of epistaxis can significantly alter the psychosocial QOL of patients [43]. Because of these irregular, spontaneous and above all unpredictable manifestations, it is essential to pay particular attention to this area that is central to QOL of HHT patients [7, 13, 44, 45].

The social relations dimension refers to the quality of relationships with family and friends in terms of communication about the disease, sharing of experiences and support. This quality of relationships has been mentioned as a source of well-being where interactions allow patients to feel supported and understood in relation to their disease [14]. Being able to share daily life with those around HHT patients could reduce certain psychosocial concerns (e.g., taboo subject, hereditary character), especially since the disease is considered by patients as an integral part of their identity [7, 42, 46–48]. Thus, this rare genetic disease highlighted that the issue of self-acceptance and acceptance of others is a key dimension of quality of (social) life [7].

The relationship with the medical profession dimension refers to the assistance provided by the caregivers. In the qualitative study conducted by Martinent et al. [14], most patients experienced feelings of helplessness and incomprehension in the face of the rarity of this genetic disease and the lack of knowledge of health professionals on the subject. Although patients are personally experts on their own disease, the involvement of caregivers in the disease would allow them to have a better relational quality, to be more involved in their care and to be more serene. In addition, one of the factors associated with the severity of epistaxis in HHT is the attention of the medical profession to the disease, so management and follow-up are aimed at avoiding the development of complications [44].

The experience of symptoms dimension represents the subjective way of living the disease. It is about how patients perceive the impact of the disease on their daily lives and adapt psychologically to the symptoms. Coping theories are useful for better understanding QOL [49]. Models of stress and coping posit that in response to a stressor, such as having a genetic problem, individuals make cognitive and emotional appraisals of that stressor [49]. These appraisals include perceptions of the personal weight of the stressor (sensitivity to the stress, its causes, its severity, its relevance to life) as well as perceptions of one's ability to cope with the problems and emotions generated by the stressor [50]. The more the individual adapts to living with the genetic disease, the better the QOL is [11].

Finally, concern about the disease evolution refers to the progression of the disease over time and the familial nature of the disease. One of the uncertainties experienced by patients concerns the evolution of the disease as it evolves silently and its complications are insidious [7]. This raises the question of the difficulties in making short, medium and long-term plans [7]. Linked to this is the feeling of anxiety and guilt associated with heredity and transmission, of seeing an elder with an unfavorable clinical course, of seeing oneself in a similar negative situation and of seeing one's children living with the disease [7, 14, 46, 47]. Thus, for many, the experience of this disease leads to apprehension with no real possibility of control.

The results of correlational analyses provided evidence of the convergent validity of the QoL-HHT scores. In addition, the pattern of correlations of QoL-HHT subscales with theoretically relevant external variables examined in the present study is consistent with the literature. In particular, our results showed that a high QOL (i.e., high scores for the dimensions of social relationships, relationship with the medical profession and experience of symptoms) was positively linked to adapted emotional regulations (e.g., positive focus, focus on action, positive reappraisal and putting it into perspective) and negatively correlated with anxiety and depression. Moreover, low QOL (i.e., high scores for the dimensions of physical limitations, concern about bleeding and concern about the evolution of the disease) was significantly and positively linked to inadequate regulation (e.g., rumination, dramatization), anxiety and depression. The scores of the generic QOL scale SF-36 was strongly related to the QOL dimensions of the QoL-HHT (except for the social dimensions (i.e., social relationships and relationship with the medical profession)), providing strong evidence for the construct validity of the QoL-HHT scores.

In France, the number of women followed in consultation for HHT disease is much higher than men, while the male/female share of the diagnosis is equal. This fact explains the higher number of women compared to men in the 2 samples of our study. Moreover, the results did not show any difference between the 2 sexes, which means that the gender factor does not impact the QOL of patient with HHT [17]. However, we can observe that women give a slightly higher importance than men regarding the relationship with medical professionals. This observation may explain the high proportion of women in medical follow-up due to a greater sensitivity and demand for a relationship with medical professionals. Lastly, this study shows an association between age and physical limitations and concern about bleeding in HHT patients. The advanced age leads to a stronger perception of one's own physical limitations due to the disease and a greater concern about nosebleeds, these 2 dimensions being themselves correlated. Several studies have shown that low QOL scores due to the disease and more particularly to epistaxis are associated with advanced age [17, 51].

The present study highlighted that factors related to health status, particularly in terms of experiencing the symptoms of the disease (especially fatigue and epistaxis), are essential to determine the QOL of patients with HHT. This confirms the results of several studies on rare genetic diseases [11, 14] as well as the statements of Patrick and Erickson [52] specifying that the value attributed to life expectancy is affected by deteriorations, states of functioning, perceptions and social opportunities that are influenced by the disease, injury, treatment and/or policy. Beyond health status, this study also investigated and confirmed the importance of psychosocial factors in determining the QOL of patients with HHT [14]. These factors refer to the subjective experience of the patients, particularly in terms of knowledge and recognition of the disease by their family, friends, professional, medical and social environment, the evolution of the disease and the questions regarding the transmission. Psychosocial factors have been highlighted to determine the QOL of people with rare genetic diseases [11].

This scale (original version validated in French in additional file 4) could be used by all practitioners receiving patients with HHT disease during consultations, during therapeutic trials or as an indirect measure of the evolution of symptoms. It would allow identifying and quantifying the aspects of the QOL more particularly impacted in HHT patients in order to be able to propose them a "tailor-made" accompaniment and/or orientation: Specialized consultations, psychologist, social worker, departmental house of the handicapped persons, drafting of mails or information documents. The scale would also make it possible to highlight the areas of the QOL (medical, physical, social, psychological) to which it is important to pay more attention in the context of the individualized follow-up of patients. In this sense, it would also be interesting to evaluate the impact and effectiveness of existing therapies on QOL with the QoL-HHT questionnaire in the follow-up of treatments for patients with HHT disease. A study showed an improvement in QOL after laser therapy of nasal telangiectasias [53].

The study has several limitations. The first is that this study was carried out only on adults, so it does not guarantee the transferability of the questionnaire to a population under 18 years old. Certainly, it would be interesting to study also the most determining dimensions in the QOL of young people with HHT disease because the QOL can be seen and experienced differently and the psychological management of the disease is probably less mature among adolescents in comparison to adults. Especially since our study shows an association between age and some dimensions related to QOL in patients with HHT. It should be noted that young people with HHT mostly experience less severe symptoms of the disease than adults but the impact of seeing disabled relatives could be burdensome. The second limitation is that we did not consider the subtypes (HHT1 and HHT2) of the disease. The symptoms differ at certain points, so it would be interesting in a future study to compare the factors related to QOL according to the types of HHT disease. The third limitation is that this study only used self-reported questionnaires. Despite its advantages in terms of ease of interpretation, low cost and speed of data collection [54], it has the disadvantage of not considering other approaches that may influence patient response such as behavioral or physiological approaches to obtain a more finely representation of the concept of QOL in HHT disease [55]. Each method should be formally established as any measurement in psychology is susceptible to bias and error [56]. Thus, further studies conducted on the HHT patients could adopt a measurement approach encompassing self-report questionnaires, physiological and behavioral measurements. Finally, it would be particularly useful to validate the QoL-HHT questionnaire in English to ensure its transferability to other non-French speaking populations.

## Conclusions

This present study identified the most determining factors in the QOL of HHT patients through the development of a questionnaire (QoL-HHT) with strong evidence for the factorial structure, reliability, and convergent validity of its scores. In agreement with theoretical frameworks and previous empirical studies, the results of the present study indicate that the QoL-HHT can be a robust measure of six key domains of HHT disease-specific QOL: Physical limitations, social relationships, concern about bleeding, relationship with the medical profession, experience of symptoms and concern about the evolution of the disease. It is a very promising tool, simple and quick self-assessment, to evaluate the impact of HHT on specific aspects of the QOL of patients in order to offer them individualized medico-psycho-social support.

## Supplementary Information

**Additional file 1.** Factor loading of the AFEs of the 78-item version of the preliminary QOL-HHT questionnaire.

**Additional file 2.** Standardized Factor Loadings (λ) and Uniqueness (δ) for Confirmatory Factor Analysis (CFA), and Exploratory Structural Equation Modeling (ESEM) of the 29-item version of the QoL-HHT questionnaire.

**Additional file 3.** Standardized factor loadings (λ) and Uniqueness (δ) for the bi-factor model of the 24- item version of the QoL-HHT questionnaire.

**Additional file 4.** The QoL-HHT questionnaire (original version validated in French).

## Data Availability

The datasets of the study are included in this published article and its supplementary files, or can be made available from the corresponding author on request.

## Abbreviations

HHT: Hereditary hemorrhagic telangiectasia
QOL: Quality of life
QoL-HHT: The quality of life questionnaire in hereditary hemorrhagic telangiectasia
SF36: Short-form 36
HAD: Hospital anxiety depressive
SSQ6: Social support questionnaire 6
CERQ: Cognitive emotional regulation questionnaire
EFA: Exploratory factorial analysis
CFA: Confirmatory factorial analysis
ESEM: Exploratory structural equation modeling
