# Supplementary material for: Development and validation of a quality of life measurement scale specific to hereditary hemorrhagic telangiectasia: the QoL-HHT
Source: Orphanet J Rare Dis. 2022 Jul 19;17:281. doi: 10.1186/s13023-022-02426-2 (PMC9295423; doi:10.1186/s13023-022-02426-2)
Supplement: Supplementary file 4 — Additional file 4. The QoL-HHT questionnaire (original version validated in French). [file 13023_2022_2426_MOESM4_ESM.pdf]

QoL – HHT

Prénom Nom ____

Date de naissance ____________________

Ce questionnaire souhaite rendre compte de votre qualité de vie. A travers cette liste de proposition il s'agit d'évaluer le retentissement de la maladie de Rendu-Osler et son importance sur votre quotidien. Pour l'ensemble des propositions suivantes nous vous remercions d'indiquer votre degré d'accord selon l'échelle allant de 1 (Pas du tout d'accord) à 5 (Tout à fait d'accord).

1 = Pas du tout d'accord / 2 = pas d'accord / 3 = ni d'accord ni pas d'accord / 4 = d'accord / 5 = tout à fait d'accord

		pas du tout d'accord				tout à fait d'accord
1	J'ai l'impression de fonctionner au ralenti	1	2	3	4	5
2	Le fait de pouvoir parler de la maladie avec ma famille m'aide à mieux la supporter	1	2	3	4	5
3	Je suis gêné(e) par le caractère soudain et imprévisible des saignements	1	2	3	4	5
4	Grâce aux conseils médicaux que l'on me propose, ma qualité de vie est préservée	1	2	3	4	5
5	Je crains que la maladie s'aggrave avec l'âge	1	2	3	4	5
6	La maladie de Rendu-Osler me limite dans mes déplacements (marche, mobilité…)	1	2	3	4	5
7	Je supporte mieux la maladie grâce au soutien apporté par mes amis	1	2	3	4	5
8	La fréquence, la durée et/ou l'intensité des saignements me dérangent beaucoup	1	2	3	4	5
9	Grâce aux recommandations médicales, je peux limiter l'impact des symptômes sur mon quotidien	1	2	3	4	5
10	Les symptômes de la maladie ne me gênent pas trop car j'ai appris à les gérer	1	2	3	4	5

		pas du tout d'accord				tout à fait d'accord
11	Je suis inquiet(e) au sujet de la santé des autres membres de ma famille qui ont la maladie de Rendu-Osler	1	2	3	4	5
12	A cause de la maladie, je suis limité(e) dans mes activités physiques intenses (courir, soulever des objets lourds, faire du sport…)	1	2	3	4	5
13	Le fait que ma famille comprenne ce dont j'ai besoin améliore mon quotidien	1	2	3	4	5
14	J'appréhende de saigner en public	1	2	3	4	5
15	La maladie de Rendu-Osler me parait tellement familière que je n'ai pas l'impression d'être malade	1	2	3	4	5
16	Je m'inquiète pour ma santé quand je vois d'autres personnes de mon entourage qui ont la maladie de Rendu-Osler	1	2	3	4	5
17	Je suis souvent fatigué(e) physiquement	1	2	3	4	5
18	Ma famille comprend ce que je vis et cela améliore mon quotidien	1	2	3	4	5
19	Je suis très embarrassé(e) de saigner en public	1	2	3	4	5
20	La qualité des relations que j'entretiens avec les soignants me permet d'être serein(e)	1	2	3	4	5
21	Je ne me sens pas gêné(e) par la maladie car j'ai l'impression d'avoir toujours vécu avec	1	2	3	4	5
22	J'appréhende l'évolution de la maladie dans le futur	1	2	3	4	5
23	Même si je saigne souvent du nez, je n'y apporte pas beaucoup d'importance car cela est devenu une habitude	1	2	3	4	5
24	Grâce aux informations données par les soignants je peux connaitre la maladie et m'impliquer dans les soins	1	2	3	4	5

Merci !
